# Renal biomarkers of acute kidney injury in response to increasing intermittent hypoxia episodes in the neonatal rat

**DOI:** 10.1186/s12882-021-02507-7

**Published:** 2021-09-04

**Authors:** Anano Zangaladze, Charles L. Cai, Matthew Marcelino, Jacob V. Aranda, Kay D. Beharry

**Affiliations:** 1 Department of Pediatrics, Division of Neonatal-Perinatal Medicine, State University of New York, Downstate Medical Center, Brooklyn, NY USA; 2 Department of Ophthalmology, State University of New York, Downstate Medical Center, Brooklyn, NY USA; 3 SUNY Eye Institute, New York, NY USA; 4 Department of Pediatrics & Ophthalmology, Neonatal-Perinatal Medicine Clinical & Translational Research Labs, State University of New York, Downstate Medical Center, 450 Clarkson Avenue, Box 49, Brooklyn, NY 11203 USA

**Keywords:** Angiotensin converting enzyme, Apoptosis, Endothelin, Neonatal intermittent hypoxia, Oxidative stress

## Abstract

**Background:**

We tested the hypotheses that: 1) early exposure to increasing episodes of clinically relevant intermittent hypoxia (IH) is detrimental to the developing kidneys; and 2) there is a critical number of daily IH episodes which will result in irreparable renal damage that may involve angiotensin (Ang) II and endothelin (ET)-1.

**Methods:**

At birth (P0), neonatal rat pups were exposed to brief IH episodes from the first day of life (P0) to P7 or from P0-P14. Pups were either euthanized immediately or placed in room air (RA) until P21. RA littermates served as controls. Kidneys were harvested at P7, P14, and P21 for histopathology; angiotensin converting enzyme (ACE), ACE-2, ET-1, big ET-1, and malondialdehyde (MDA) levels; immunoreactivity of ACE, ACE-2, ET-1, ET-2, ET receptors (ET_A_R, ET_B_R), and hypoxia inducible factor (HIF)_1α_; and apoptosis (TUNEL stain).

**Results:**

Histopathology showed increased renal damage with 8–12 IH episodes/day, and was associated with Ang II, ACE, HIF_1α_, and apoptosis. ACE-2 was not expressed at P7, and minimally increased at P14. However, a robust ACE-2 response was seen during recovery with maximum levels noted in the groups recovering from 8 IH episodes/day. ET-1, big ET-1, ET_A_R, ET_B_R, and MDA increased with increasing levels of neonatal IH.

**Conclusions:**

Chronic neonatal IH causes severe damage to the developing kidney with associated elevations in vasoconstrictors, suggesting hypertension, particularly with 8 neonatal IH episodes. ACE-2 is not activated in early postnatal life, and this may contribute to IH-induced vasoconstriction. Therapeutic targeting of ACE and ET-1 may help decrease the risk for kidney injury in the developing neonate to prevent and/or treat neonatal acute kidney injury and/or chronic kidney disease.

**Supplementary Information:**

The online version contains supplementary material available at 10.1186/s12882-021-02507-7.

## Introduction

Hypoxemic respiratory failure requiring mechanical ventilation occurs in approximately 18 per 1000 live births [1], and is associated with increased risk of mortality and morbidity [2]. Extremely low gestational age neonates (ELGANs) born at < 28 weeks gestation with hypoxemic respiratory failure frequently experience arterial oxygen desaturations, apneas, or intermittent hypoxia (IH), lasting < 3 min in duration [3]. Resolving hypoxic/apneic events are often accompanied by increasing the fraction of inspired oxygen (FIO_2_) resulting in a transient period of hyperoxia overshoot [4]. These IH episodes are due to immature respiratory systems, cessation of respiratory neural output [5], low blood oxygen capacity, blood volume, and hemoglobin content [6]. Studies show that the number of IH episodes are significantly high (50–100/day) during the first few weeks of life [4]. Chronic exposure to supraphysiological oxygen and neonatal IH can have deleterious effects on the immature kidneys, increasing the risk of renal diseases during adulthood [7].

Nephrogenesis occurs at the time of birth for the majority of preterm infants, but whether postnatal renal development follows a similar trajectory to normal in utero growth is unknown [8, 9]. Many preterm infants are at higher risk for renal issues including acute kidney injury and chronic kidney disease [10, 11]. Studies comparing the tissues of kidneys from preterm neonates to term controls found that renal maturation accelerated after preterm birth, with an increased number of glomerular generations [12]. However, compared with gestational controls, preterm kidneys had a greater percentage of morphologically abnormal glomeruli and a significantly larger cross-sectional area of the renal corpuscle, suggestive of renal hyperfiltration [12]. These observations suggested that the preterm kidney may have fewer functional nephrons increasing the vulnerability to impaired renal function in both the early postnatal period and later in life. Moreover, birth weight may be directly correlated the number of glomeruli [12–14].

Acute kidney injury (AKI), commonly seen in premature neonates in the intensive care unit (ICU), has been associated with later development of chronic kidney disease (CKD), hypertension and proteinuria [13–16]. Reduced nephron numbers may predispose ELGANs to hypertension because of a reduced filtration surface area, which may limit sodium excretion and would be less likely to meet increased functional demands of the body. Neonatal IH, combined with reduce renal function may further exacerbate kidney damage, and impact key pathways which may later contribute to the development of chronic kidney disease and hypertension. Although the factors that may contribute to acute kidney injury have been extensively studied [15–18], there is a paucity of information with regard to the effects of neonatal IH on the developing premature kidneys.

The renin-angiotensin system (RAS) is a key regulator of blood pressure, fluid/electrolyte homeostasis, and kidney development. The major hormone generated in the RAS system is angiotensin II (Ang II) which directly constricts vascular smooth muscle cells, stimulates aldosterone production, and increases sodium reabsorption, contributing to hypertension [19]. Angiotensin converting enzyme (ACE) generates the vasoactive Ang II from the inactive precursor Ang I [20], and hence, ACE inhibitors are effective and widely used for the treatment of hypertension and kidney diseases. Conversely, ACE-2 hydrolyzes Ang II with high efficiency, resulting in opposing actions to Ang II, including release of vasodilatory products such as nitric oxide (NO), prostaglandin E_2_, and bradykinin [21]. Endothelin (ET-1) is a 21 amino acid peptide that is considered the most potent vasoconstrictor in the human body. Formation of ET-1 occurs by cleaving of preproET-1 to the biologically inactive big ET-1 (38 amino acids). Endothelin converting enzyme then splits big ET-1 to the biologically active ET-1 [22]. ET-1 is produced by almost every cell type in the kidney, but predominantly by endothelial and tubular cells. ET-1 exerts its actions by binding to specific ET_A_ and ET_B_ receptors [23]. ET-1 signaling to ET_A_R mediates vasoconstriction, while signaling to ET_B_R results in vasodilation via increased prostacyclin and NO [24]. An imbalance between ET_A_R and ET_B_R activity leads to renal dysfunction and disease [25].

We previously showed that clustered IH events were associated with a more severe outcome in oxygen-induced retinopathy than dispersed IH events [26]. This was later proven in preterm infants [27]. Using the same rat model of clustered neonatal IH [26], which simulates IH experienced by ELGANs, we tested the hypotheses that: 1) early exposure to increasing episodes of neonatal IH has detrimental effects on the developing kidneys; and 2) that there is a critical number of daily IH episodes which will result in irreparable renal damage that may involve Ang II and ET-1. Our hypotheses were tested to: 1) examine the effects of increasing episodes of neonatal IH on renal histopathology and morphometry (primary objective); 2) determine whether IH-induced renal damage is associated with Ang II, ET-1, and biomarkers of hypoxia and oxidative stress; and 3) identify the critical number of neonatal IH episodes that induce Ang II, ET-1, biomarkers of hypoxia and oxidative stress, and renal damage (secondary objectives).

## Material and methods

### Ethical statement

All procedures performed in this study were conducted in accordance with the ethical standards of the institutional animal care and use committee (IACUC approval number 11–10255) of SUNY Downstate Medical Center; the Guide for the Care and Use of Laboratory Animals (National Research Council); the United States Department of Agriculture; and in compliance with the Animal Research: Reporting of In Vivo Experiments (ARRIVE) guidelines. Animals were euthanized according to the guidelines of the American Veterinary Medical Association (AVMA) Panel for euthanasia of animals (Ver. 2020). All necessary measures to reduce pain and distress before and during euthanasia were applied.

### Animals

Certified infection-free, timed-pregnant Sprague Dawley rats were purchased from Charles River Laboratories (Wilmington, MA) at 17 days gestation. The animals were housed in an animal facility with a 12-h-day/12-h-night cycle and provided standard laboratory diet and water ad libitum until delivery of their pups at 22 days gestation. Within 2–3 h of birth, newborn rat pups delivering on the same day were pooled and randomly assigned to expanded litters of 18 pups/litter (9 males and 9 females). Animals with a birth weight > 10% below or above the mean birth weight were excluded. Sex was determined by the anogenital distance. Each pup was weighed and measured for linear growth (crown to rump length in centimeters), as previously described [28].

### Sample size

A sample size of 18 pups was used to simulate poor nutrition of preterm infants. We used GraphPad Statmate (GraphPad, San Diego, CA, USA) for sample size determination. The sample size calculations were based on previous data comparing means from unpaired t-test. A sample size of 18 pups in each group was calculated to have an 80% power to detect a difference between means with a significance level (alpha) of 0.05 (two-tailed). This sample size has been shown to produce oxidative stress in neonatal rats [28].

### Experimental design

A total of 31 groups of 18 rat pups (9 males and 9 females) were studied according to the experimental design [28]. Briefly, there were 3 normoxia groups (P7, P14, and P21), 4 hyperoxia (50%O_2_) groups (P7, P14, P21–7, and P21–14), 4 groups each for 2, 4, 6, 8, 10, and 12 IH episodes (P7, P14, P21–7, and P21–14) for a total of 24 IH groups. Groups assigned to P21–7 were exposed to IH for 7 days and recovery/oxygenation in normoxia for 14 days. Groups assigned to P21–14 were exposed to IH for 14 days and recovery/oxygenation in normoxia for 7 days (Supplemental Figure S1). These groups were euthanized on P21. The total number of animals used for these experiments was 558. Two groups of animals served as control: 1) 50% O_2_ only with no IH events; and 2) room air (RA) only. Pups remained undisturbed in each oxygen environment until the time of euthanasia at P7, P14, and P21, except for bedding and water changes every 48 h. Animals were monitored twice daily. No unexpected adverse events were noted. At P7, P14, and P21 animals were euthanized by decapitation without anesthesia. Since these are experiments involve intermittent hypoxia and hyperoxia, this method was used to prevent the known effects of anesthesia and/or carbon dioxide inhalation on hypoxia production.

### Intermittent hypoxia (IH) cycling

The IH cycles consisted of hyperoxia (50% O_2_)/hypoxia (12% O_2_) in stepwise increments of brief (1-min), hypoxia (12%) clusters (3 clusters) during 50% O_2_. The number of IH episodes per day increased incrementally from 2 episodes/day to 12 episodes/day as previously described [28], and as presented in the Supplemental Figure (S2).

### Sample collection

Whole kidneys were harvested, rinsed in ice-cold phosphate buffered saline (pH 7.4) on ice, and weighed. For histopathology, kidneys were placed in 10% neutral buffered formalin (NBF). For ELISA assays, 5 mg tissue were placed in separate Lysing matrix D tubes with ceramic beads (MP Biomedicals, LLC, Irvine CA, USA), snap frozen in liquid nitrogen, and stored at -80 °C. Unstained paraffin-embedded sections of the kidneys were used for immunohistochemistry (IHC) of ACE, ACE-2, ET_A_R, and ET_B_R, HIF_1α_, and TUNEL assay.

### Histopathology

Whole kidneys fixed in 10% NBF were sent to the Pathology Department of State University of New York (SUNY) Downstate Medical Center for processing, embedding, and sectioning (5 μm thickness). The sections were stained with hematoxylin and eosin (H&E) according to standard laboratory techniques. Sections were imaged using an Olympus BX53 microscope, DP72 digital camera, (Olympus, Center Valley, PA USA), attached to a Dell Precision T3500 computer (Dell, Round Rock, TX USA), and analyzed using the CellSens software (Olympus America, Center Valley, PA, USA). Morphometric analyses, including kidney weight, length, width, and glomerular number, circumference, and diameter, were determined using the count and measure tool of the CellSens software (Olympus America, Center Valley, PA, USA). Images were captured at 20X magnification (1600 × 1200 pixels; scale bar = 50 μm). Resolution was enhanced using Adobe Photo Shop (San Jose, CA USA).

### ELISA assays

Lysing Matrix D tubes containing kidney samples and sterile phosphate buffered saline (PBS) were placed in a Fast Prep 24 instrument (MP Biomedicals, LLC, Irvine CA, USA) and homogenized. The homogenates were centrifuged at 10,000 rpm at 4 °C and the supernatant transferred to a clean Eppendorf tube. The samples were filtered and the filtrate was used for determination of ACE, ACE-2, ET-1, and big ET-1 using rat enzyme-linked immunosorbent assay (ELISA) kits purchased from MyBiosource, San Diego, CA, USA. A portion of the filtrate was used to determine total cellular protein levels.

### Malondialdehyde (MDA) assay

MDA levels (biomarker for lipid peroxidation) in the kidney homogenates were determined in using commercially available kits purchased from Millipore-Sigma (St. Louis, MO, USA). Samples were processed and assayed according to the manufacturer’s protocol. A total of 6 samples per experimental group were analyzed. All data were standardized using total cellular protein levels.

### Total cellular protein assay

On the day of the assay, renal homogenates were assayed for total protein levels using the dye-binding Bio-Rad protein assay (Bio-Rad, Hercules, CA) with bovine serum albumin as a standard.

### Immunohistochemistry (IHC)

Unstained paraffin-embedded kidney sections were treated with xylenes and alcohols to remove the paraffin. After unmasking the antigens, the sections were blocked with blocking buffer for 1 h and incubated overnight with primary antibodies (1:1000 dilution) against ACE (#SC12187), ACE-2 (#SC20998), ET-1 (#SC21625), ET-2 #(SC21627), HIF_1α_ (#SC10790), ET_A_R, (#PIPA3065), ET_B_R (#PIPA3066), from Santa Cruz Biotechnologies (Dallas, TX, USA), and Invitrogen (Suwanee, GA, USA), respectively. After washing with PBS, the sections were incubated with Cell Signaling (Danvers, MA, USA) Signal Stain Boost IHC reagent (HRP-conjugated), washed, mounted with Prolong Antifade, imaged and quantified using the CellSens software. Quantitative analysis was determined using the count and measure tool of the CellSens software (Olympus America, Center Valley, PA, USA). Images were captured at 20X magnification (1600 × 1200 pixels; scale bar = 50 μm). Resolution was enhanced using Adobe Photo Shop (San Jose, CA USA).

### Apoptosis (TUNEL stain)

Apoptosis was determined in the kidney sections using the TUNEL assay kit purchased from Abcam (Waltham, MA, USA), according to the manufacturer’s protocol. Sections were counterstained with Methyl Green. Images were captured at 40X magnification (1600 × 1200 pixels; scale bar = 20 μm). Quantitative analysis was determined using the count and measure tool of the CellSens software (Olympus America, Center Valley, PA, USA). Resolution was enhanced using Adobe Photo Shop (San Jose, CA USA).

### Statistical analysis

To determine differences among the groups, a test for normality of variances was conducted using the Bartlett’s test. Normally distributed data were analyzed using one-way analysis of variance (ANOVA) with Dunnett’s post-hoc tests. Non-normally distributed data were analyzed using Kruskall Wallis test with Dunn’s multiple comparison test. Data are presented as mean ± SEM and a *p*-value of < 0.05 was considered as statistically significant, using SPSS version 16.0 (SPSS Inc., Chicago, IL, USA). Graphs were prepared using GraphPad Prism version 7.03 (GraphPad, San Diego, CA, USA).

## Results

### Kidney weights

**Fig. 1:**
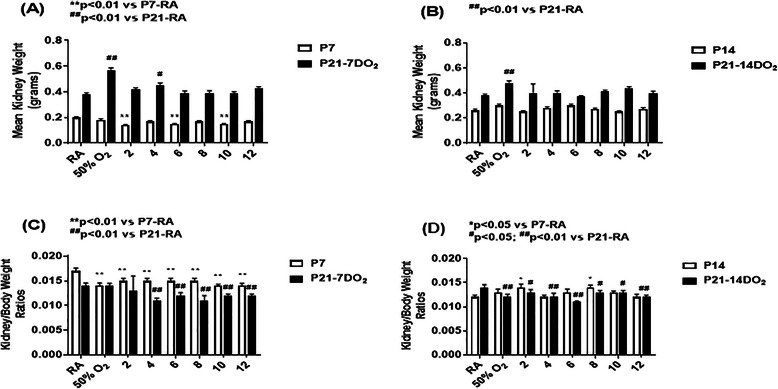
Effects of hyperoxia and incremental IH episodes on kidney weights (panels **A** and **B**) and kidney/body weight ratios (panels **C** and **D**) in neonatal rats at P7 or P14 (open bar) and P21 (solid bar). P7 animals were exposed to brief clustered IH episodes during hyperoxia (50% O_2_) or constant 50% O_2_ from P0 to P7. P14 animals were exposed to brief clustered IH episodes during 50% O_2_ or constant 50% O_2_ from P0 to P14. P21 animals were exposed to constant 50% O_2_ or brief clustered IH episodes during 50% O_2_ from P0 to P7 or P0 to P14 then placed in RA from P7 to P21 or P14 to P21 for re-oxygenation. Control animals were raised in room air (RA) from P0-P7, P0-P14 or P0-P21. Data are presented as mean ± SEM (*n* = 18 samples/group)

Mean kidney weights (panels A and B) and kidney/body weight ratios (panels C and D) are presented in Fig. 1. At P7 (panel A), mean kidney weights were lower in the 2, 6 and 10 IH groups compared to RA. At P21 following 7 days of IH and recovery in RA for 14 days (P21–7DO_2_), mean kidney weights increased in the hyperoxia and 4 IH groups. Mean kidney weights in all the oxygen groups were comparable at P14 compared to RA. At P21, only the hyperoxia group resulted in higher mean kidney weights (panel B). Kidney/body weight ratios at P7 (panel C) were significantly lower in all oxygen groups than RA. The P21–7DO_2_ groups showed reductions with 4–12 IH episodes. At P14 (panel C), kidney/body weight ratios rebounded with 2 and 8 IH episodes. However, reductions were sustained in all the P21–14DO_2_ groups (panel D). Comparisons between sex revealed significant reductions in kidney weights (0.36 ± 0.018, *p* < 0.01 vs. 0.44 ± 0.02) and kidney/body weight ratios (0.01 ± 0.0008 vs. 0.015 ± 0.0009) in males in the group exposed to 4 IH episodes (P21–14DO_2_) only.

### Histopathology and morphometry

**Fig. 2:**
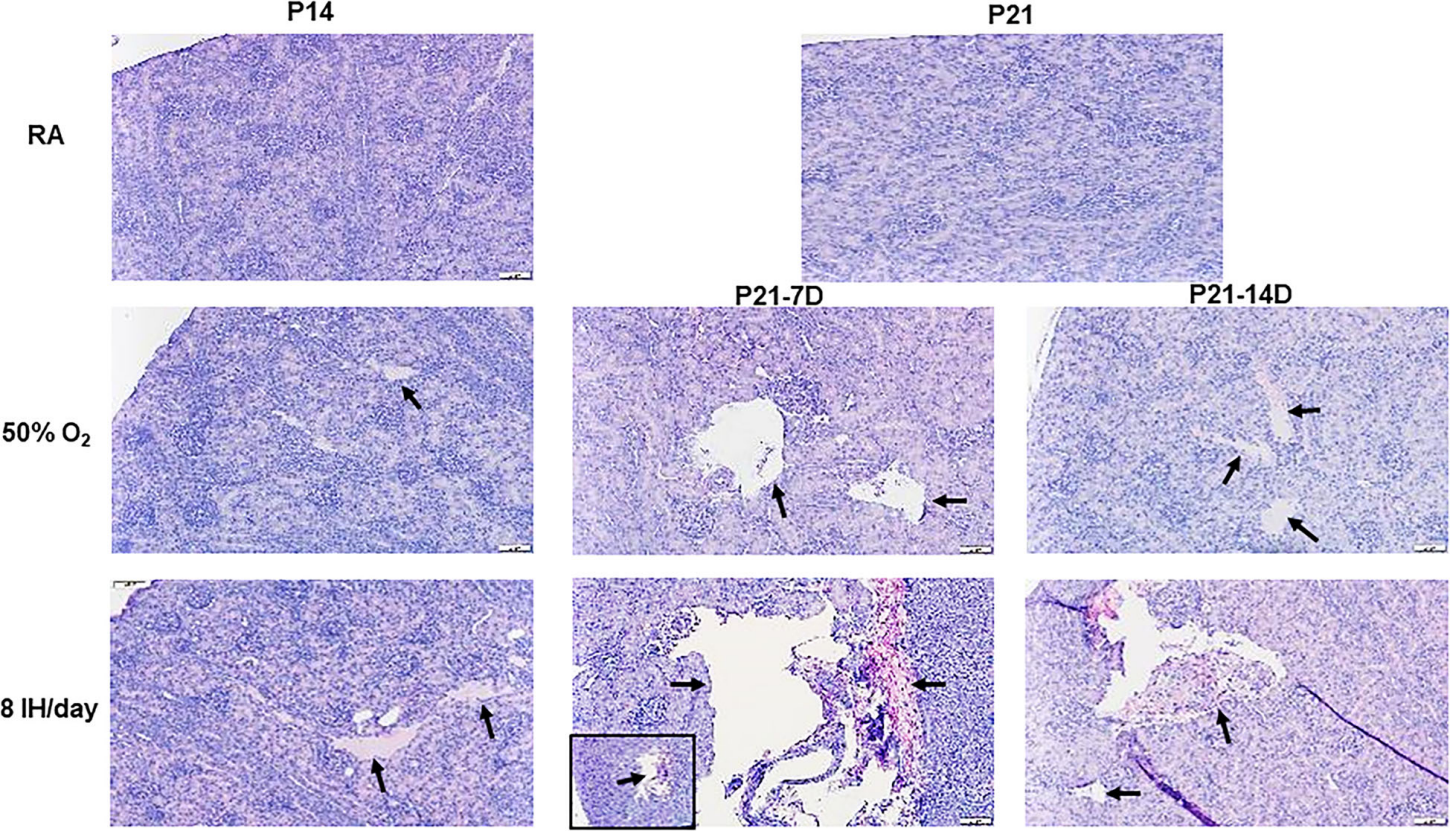
Representative H&E stained renal cortex from animals exposed to RA, hyperoxia (50% O2) or neonatal IH (8 episodes/day) at P14 or P21. P21–7D groups were exposed to hyperoxia or neonatal IH from P0-P7, then placed in RA from P7-P21. P21–14D groups were exposed to hyperoxia or neonatal IH from P0-P14, then placed in RA from P14-P21. Data shows severe damage in the groups recovering from 8 IH episodes per day (arrows). Images are 20X magnification (scale bar is 50 μm)

**Fig. 3:**
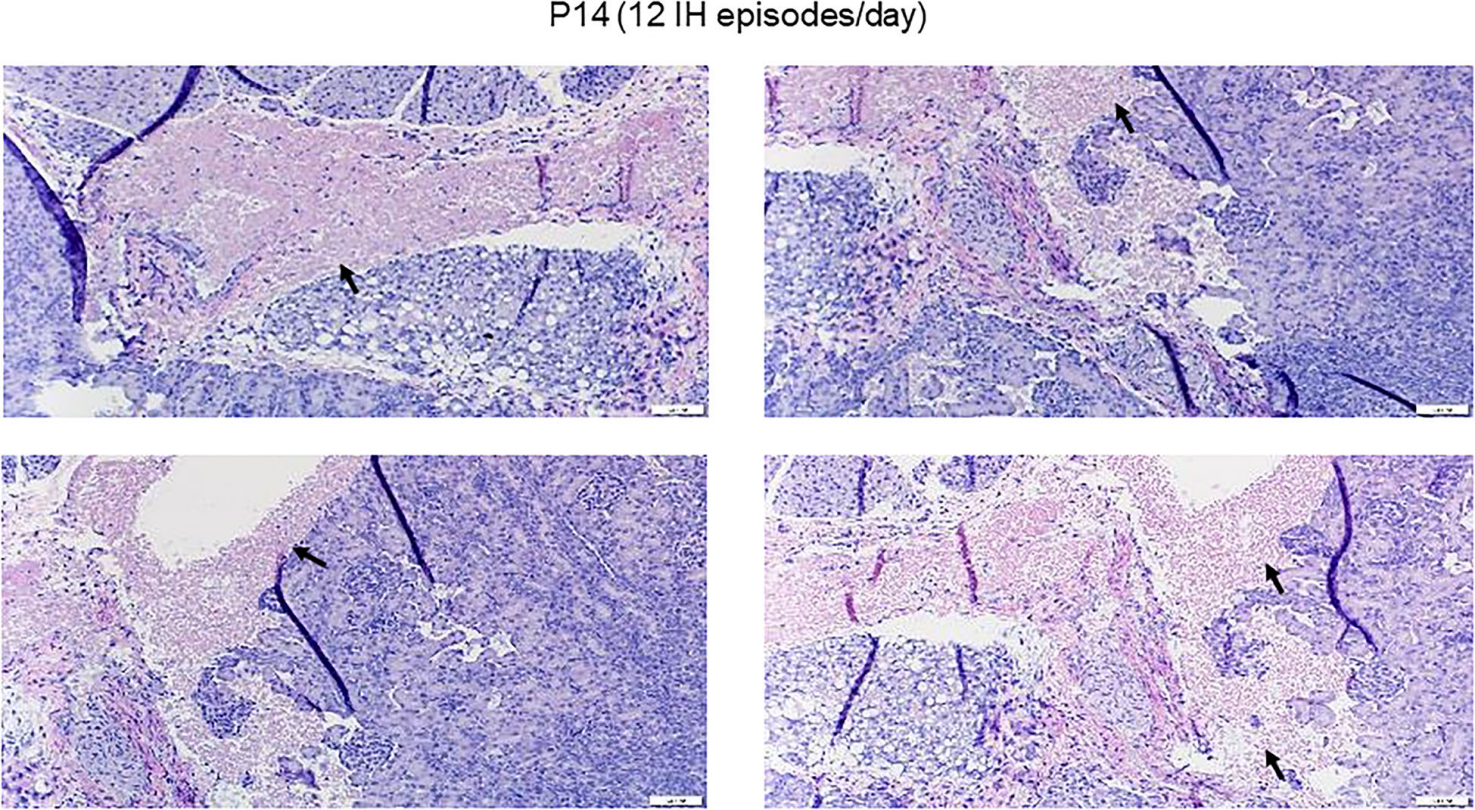
Representative H&E stained renal cortex from animals exposed to 12 neonatal IH episodes/day for 14 days. Data shows severe damage represented by massive hemorrhage and apoptosis (arrows). Images are 40X magnification (scale bar is 20 μm)

Figures 2 and 3 represents histopathology of the renal cortex. Only samples demonstrating damage are shown. Exposure to 50% O_2_ for 14 days (P14) showed the appearance of necrosis (arrow). During the recovery/reoxygenation period (P21–7DO_2_) irregular vacuolizations developed secondary to ischemia (arrows). Exposure to 50% O_2_ for 14 days with 7 days of recovery (P21–14DO_2_) showed reduced numbers of glomeruli and necrosis (arrows). Exposure to neonatal IH for 14 days without recovery/reoxygenation showed more severe necrosis (arrows). Exposure to 8 neonatal IH episodes for 7 and 14 days with recovery/reoxygenation resulted in severe tissue damage and hemorrhage (arrows). There was also notable breakdown of the glomeruli capsule. Figure 3 shows the samples that were exposed to 12 neonatal IH episodes for P14 without recovery/reoxygenation. There was significant and pervasive hemorrhage (arrows), as well as breakdown of glomeruli and tubule structure. Compared to RA, exposure to 50% O_2_ for 14 days resulted in increased kidney size (4221.4 ± 174.8, *p* < 0.01 vs. 2152.0 ± 198.5) and reduction in the number of glomeruli (8.6 ± 0.55, *p* < 0.01 vs 13.6 ± 1.8). Recovery from 50% O_2_ for 7 days caused a further increase in kidney size (5557.3 ± 219.5, *p* < 0.01 vs. 4160.7 ± 93.8) and reduction in the number of glomeruli (5.8 ± 1.1 vs. 10.0 ± 0.68). Recovery from 50% O_2_ for 14 days reduced kidney to body weight ratios and number of glomeruli (5.6 ± 0.74 vs. 10.0 ± 0.68), but did not affect kidney size. Exposure to neonatal IH (8 episodes/day) increased kidney to body weight ratios, but had no effect on kidney size or number of glomeruli. However, recovery from neonatal IH for 7 and 14 days decreased kidney to body weight ratios, but reductions in kidney size (3374.2 ± 149.2, *p* < 0.01) and number of glomeruli (5.6 ± 0.89, *p* < 0.01 vs. 10.0 ± 0.68) were noted only with 14-day recovery group.

### Effect on lipid peroxidation

**Fig. 4:**
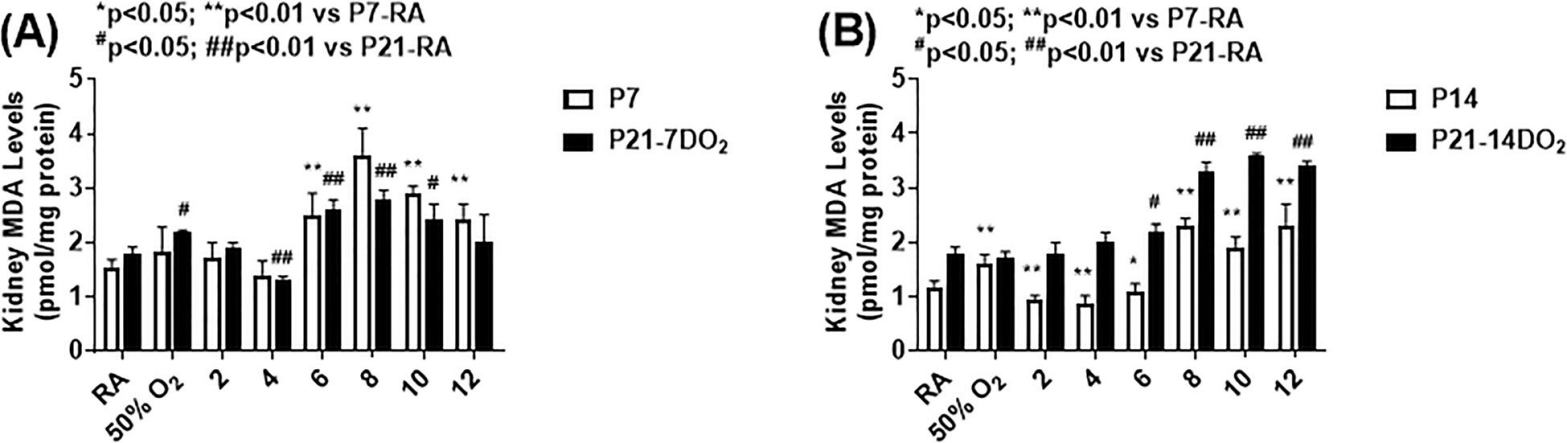
Effects of hyperoxia and incremental IH episodes on malondialdehyde (MDA) levels in the kidney homogenates from neonatal rats at P7 or P14 (open bar) and P21 (solid bar). Panel **A** shows the groups exposed from P0-P7 and P0-P7 with recovery/reoxygenation for 14 days from P7-P21. Panel **B** shows the groups exposed from P0-P14 and P0-P14 with recovery/reoxygenation for 7 days from P14-P21. Groups are as described in Fig. 1. Data are presented as mean ± SEM (*n* = 6 samples/groups)

Figure 4 shows MDA levels in the kidney homogenates. Panel A represents in the groups exposed to hyperoxia or neonatal IH for 7 days (white bar) or 7 days with recovery in RA for 14 days (black bar), and panel B represents the groups exposed to hyperoxia or neonatal IH for 14 days (white bar) or 14 days with 7 days of recovery in RA. Exposure to 6–12 IH episodes for 7 days induced MDA levels, as did recovery from hyperoxia and recovery from 6 to 10 IH episodes (panel A). Exposure for 14 days resulted in higher levels in the group exposed to hyperoxia, but decreased MDA levels with 2–6 IH episodes. A progressive increase in MDA was noted in the groups exposed to 8–12 IH episodes. MDA was robustly elevated in the groups recovering from 6 to 12 IH episodes (panel B).

### Effect on ANG II

**Fig. 5:**
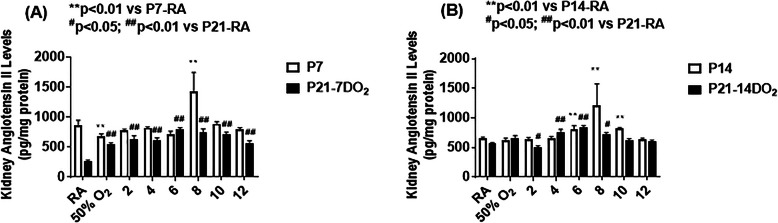
Effects of hyperoxia and incremental IH episodes on angiotensin II (Ang II) levels in the kidney homogenates from neonatal rats at P7 or P14 (open bar) and P21 (solid bar). Panel **A** shows the groups exposed from P0-P7 and P0-P7 with recovery/reoxygenation for 14 days from P7-P21. Panel **B** shows the groups exposed from P0-P14 and P0-P14 with recovery/reoxygenation for 7 days from P14-P21. Groups are as described in Fig. 1. Data are presented as mean ± SEM (*n* = 6 samples/groups)

Figure 5 shows the effects of hyperoxia and neonatal IH on renal levels of ANG II. Panel A shows data from animals exposed for 7 days (white bar) or exposure for 7 days with recovery for 14 days (black bar). Panel B shows data from animals exposed for 14 days (white bar) or exposure for 14 days with recovery for 7 days (black bar). At P7, ANG II was reduced with hyperoxia and 8 neonatal IH episodes/day compared to age-matched control groups in RA (panel A). Conversely, at P21, ANG II levels were elevated in all neonatal IH groups during the recovery/reoxygenation period compared to RA counterparts. There was a progressive increase from 0 to 4, a peaked with 6–10, then a decline at 12, neonatal IH episodes/day. Exposure for 14 days resulted in elevations in ANG II with 6, 8, and 10 neonatal IH episodes/day with the highest elevations noted in the 8/day group. Similar elevations were noted in the groups recovering from 4, 6, and 8 neonatal IH episodes/day, although a decline was seen with 2/day (panel B).

### Effect on ACE and ACE-2

**Fig. 6:**
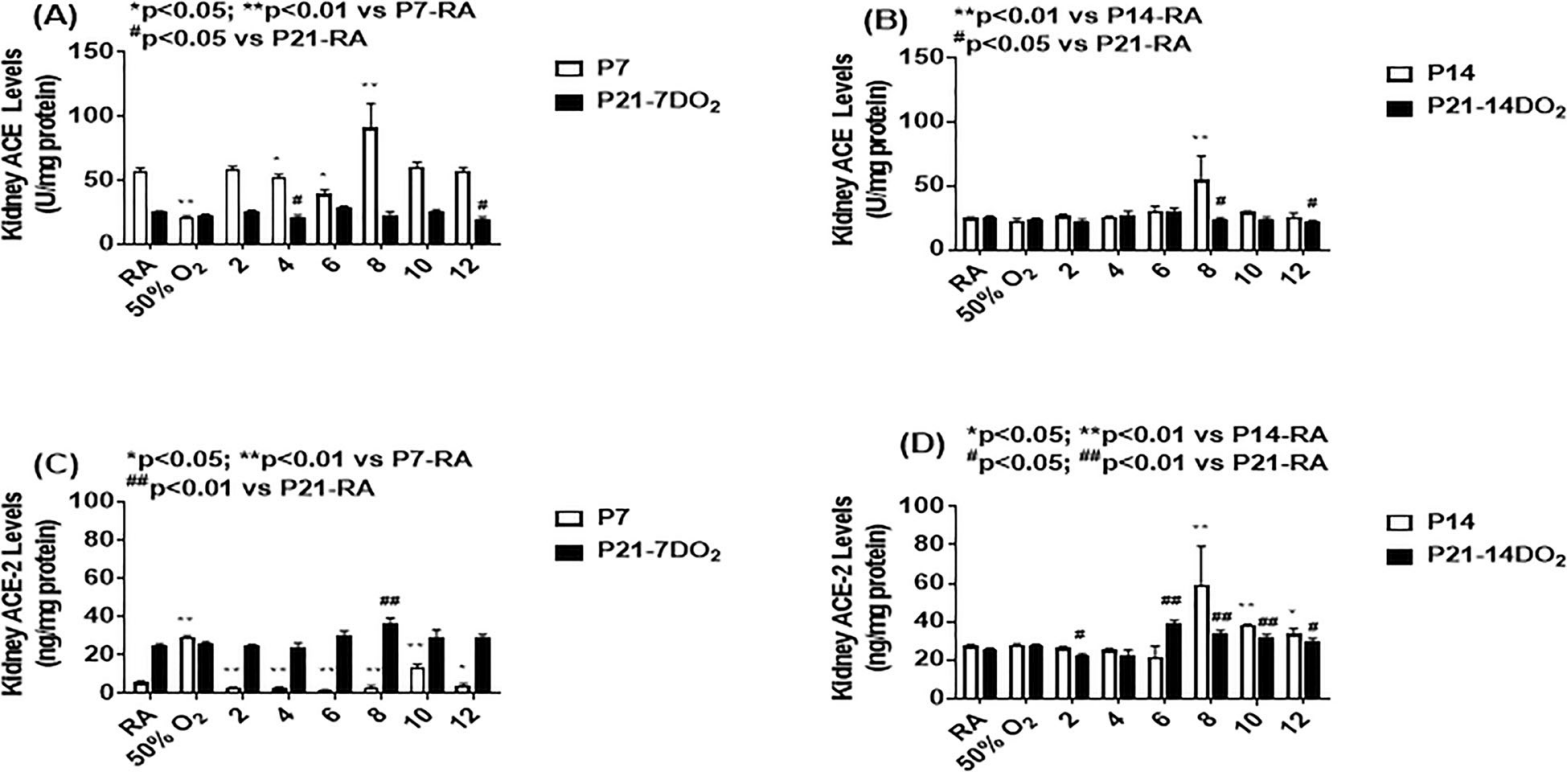
Effects of hyperoxia and incremental IH episodes on ACE (panels **A** and **B**) and ACE-2 (panels **C** and **D**) levels in the kidney homogenates from neonatal rats at P7 or P14 (open bar) and P21 (solid bar). Panels **A** and **C** are groups exposed from P0-P7 and P0-P7 with recovery/reoxygenation for 14 days from P7-P21. Panels **B** and **D** are groups exposed from P0-P14 and P0-P14 with recovery/reoxygenation for 7 days from P14-P21. Groups are as described in Fig. 1. Data are presented as mean ± SEM (*n* = 6 samples/groups)

**Fig. 7:**
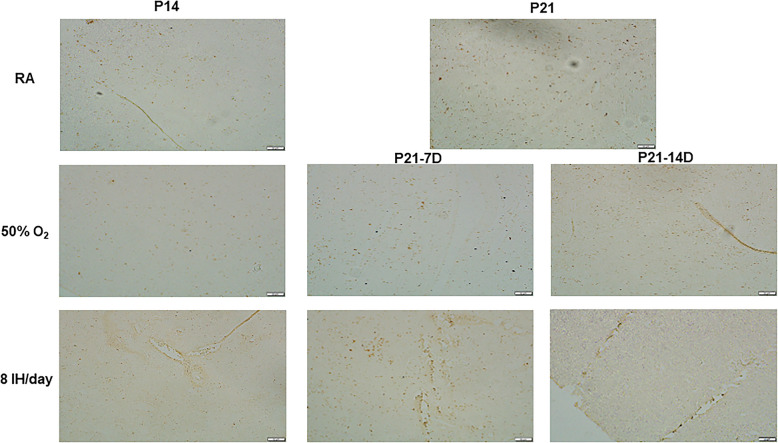
Representative immunohistochemistry staining of ACE in the kidneys in response to hyperoxia and IH episodes (8 episodes/day) at P14 and P21. P21–7D groups were exposed to hyperoxia or neonatal IH from P0-P7, then placed in RA from P7-P21. P21–14D groups were exposed to hyperoxia or neonatal IH from P0-P14, then placed in RA from P14-P21. Images are 20X magnification (scale bar is 50 μm)

**Fig. 8:**
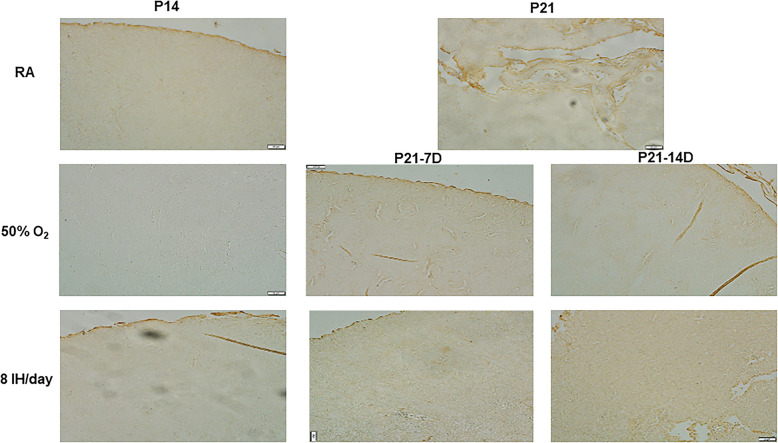
Representative immunohistochemistry staining of ACE-2 in the kidneys in response to hyperoxia and IH episodes (8 episodes/day) at P14 and P21. P21–7D groups were exposed to hyperoxia or neonatal IH from P0-P7, then placed in RA from P7-P21. P21–14D groups were exposed to hyperoxia or neonatal IH from P0-P14, then placed in RA from P14-P21. Data shows severe damage in the groups recovering from 8 IH episodes per day (arrows). Images are 20X magnification (scale bar is 50 μm)

The effects of increasing episodes of neonatal IH on ACE (panels A and B) and ACE-2 (panels C and D) in the kidney homogenates are presented in Fig. 6. Exposure to hyperoxia for 7 days decreased ACE levels as did exposure to 4 and 6 neonatal IH episodes/day. In contrast, exposure to 8/day caused a significant elevation in ACE. During the recovery/reoxygenation period, only 4 and 12 episodes/day caused a decline in ACE levels (panel A). Exposure to 8 IH episodes/day for 14 days resulted in elevations in ACE, and recovery/reoxygenation from 8 and 12 episodes/day decreased it (panel B). Neonatal IH had the most significant effect on ACE-2 particularly in the groups exposed for 7 days. While hyperoxia increased ACE-2, levels declined substantially in all neonatal IH groups. During the recovery/reoxygenation period, levels were elevated only in the 8/day group (panel C). Conversely, exposure for 14 days caused an opposite elevation with 8–12 IH episodes/day. Levels remained elevated in the groups exposed to 6–12 episodes/day despite recovery/reoxygenation, although levels declined in the group recovering from 2 episodes/day (panel D). Figure 7 shows immunoreactivity of ACE in the renal cortex. Robust staining was seen in the group recovering from 14 days of hyperoxia (P21–14DO_2_) and in all groups exposed to 8 IH episodes/day. Figure 8 shows the immunoreactivity of ACE-2. ACE-2 was highly expressed in the RA groups. Exposure to hyperoxia for 14 days decreased ACE-2 expression, an effect that rebounded with 14 and 7 days of recovery/reoxygenation in RA. Exposure to 8 neonatal IH episodes had no effect on ACE-2, supporting Fig. 6. Table 1 shows the quantitative assessment of the intensity of ACE and ACE-2 immunoreactivity in the samples. Compared to RA, exposure to hyperoxia and neonatal IH increased ACE and ACE-2 expression in all groups, although the highest expression was seen with neonatal IH (8 episodes/day), correlating with the ELISA results.

**Table 1:** Quantitative analysis of immunostaining intensities

Variable	ACE (***n*** = 6)	ACE2 (***n*** = 6)	ET-1 (***n*** = 6)	ET-2 (***n*** = 6)	ET_**A**_ (***n*** = 6)	ET_**B**_ (***n*** = 6)	HIF_**1α**_ (***n*** = 12)	TUNEL (***n*** = 12)
***Room Air (RA)***								
P14	1161.8± 173.3	1671.0± 566.1	1877.8± 429.7	8254.0± 353.4	2988.5± 396.7	3255.3± 504.2	388.2 ± 60	105.8 ± 15.6
P21	1968.8± 171.8	4523.3± 434.3	1295.8± 35.9	3922.0± 795.1	2652.7± 410.7	3558.5± 310.2	246.7 ± 50	107.7 ± 23.2
***50% O2***								
P14	3123.6± 426.0*	1117.0± 32.8	10,158.8± 2408.4**	8041.8± 1998.2	6863.2± 224.5*	951.8± 84.5*	554.0± 136.8	5291.1± 180.6**
P21–7D	2077.2± 189.3	17,920.6± 2485.8**	17,723.5± 3088.7**	2266.8± 496.0	370.8± 38.5**	220.0± 39.5**	5381.0± 1085.6**	3746.2± 490.0**
P21–14D	11,390.7 ± 2356.4*	19,296.2± 2904.2**	12,053.4 ± 1790.1**	8720.2± 798.8**	1649.7± 73.0*	1849.8± 243.9**	4978.7± 1025.4**	3625.0± 723.9**
***Neonatal Intermittent Hypoxia (8 episodes/day)***								
P14	3874.0± 816.5**	6731.5± 780.8**	26,629.0± 1495.3**	6473.0± 1333.2	11,053.0± 1523.2**	17,013± 924.4**	4596.2± 959.6**	8191.6± 634.9**
P21–7D	18,342.0 ± 1835.9**	45,587.2± 2696.8**	30,999.3± 4202.0**	40,048.3 ± 5332.7**	4065.0± 246.6**	1453.8± 167.5**	4775.8± 200.4**	2336.4± 605.6**
P21–14D	27,158.0 ± 4041.5**	37,150.7 ± 1561.8**	15,976.4 ± 1611.9**	2207.6± 442.9	2487.2± 111.7	1652.6± 133.2**	11,571.2 ± 3812.0**	3533.0± 152.8**

Data are mean ± SEM (*n* = number/group; *p < 0.05; **p < 0.01 vs RA). Comparison among the groups for each age category was determined by one-way ANOVA with Dunnett’s multiple comparison post hoc test. Animals were exposed to 14 days of neonatal IH (P14), or placed in room air for recovery for 14 days after 7 days of neonatal IH (P21–7D), or for 7 days after 14 days of neonatal IH (P21–14D)

### ACE/ACE-2 ratios

**Fig. 9:**
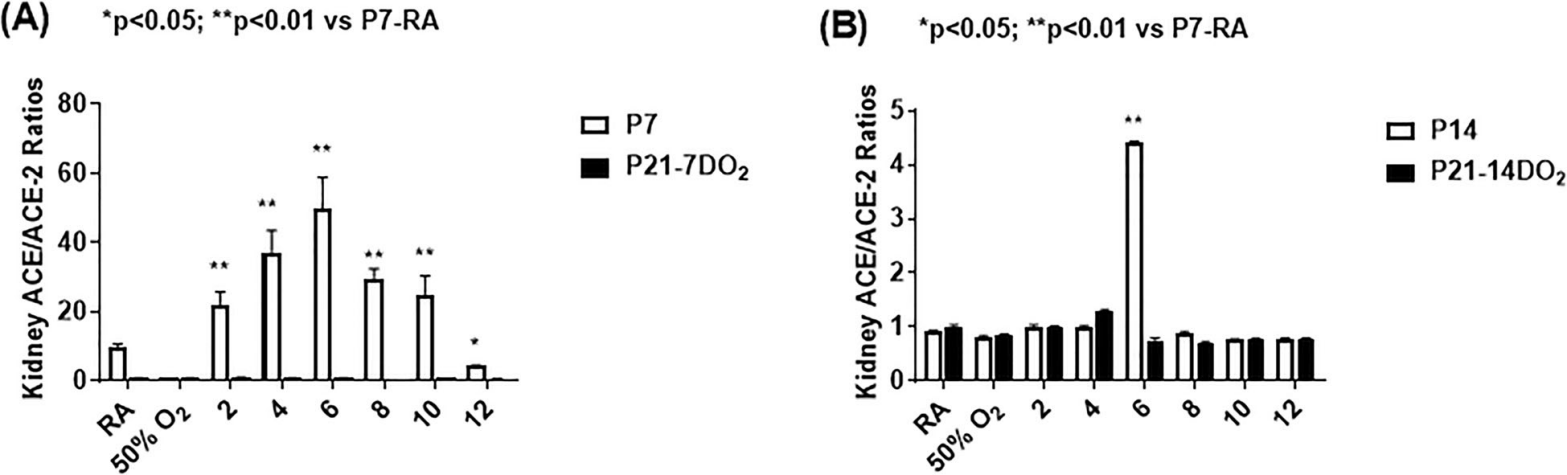
Effects of incremental IH episodes on ACE/ACE-2 ratios in the kidney homogenates from neonatal rats at P7 or P14 (open bar) and P21 (solid bar). Panel **A** shows the groups exposed from P0-P7 and P0-P7 with recovery/reoxygenation for 14 days from P7-P21. Panel **B** shows the groups exposed from P0-P14 and P0-P14 with recovery/reoxygenation for 7 days from P14-P21. Groups are as described in Fig. 1. Data are presented as mean ± SEM (*n* = 6 samples/groups)

Figure 9 shows the ratio of ACE/ACE-2 levels following 7 days (panel A) or 14 days (panel B) of hyperoxia or neonatal IH exposure. In the groups exposed for 7 days, the ratio of ACE/ACE-2 showed an interesting palindromic curve with progressive incremental increases that peaked with 6 IH episodes/day followed by an obverse decline with 8–12 episodes/day. No differences were noted among the recovery/reoxygenation groups (panel A). In the groups exposed for 14 days exposure to 6 episodes/day resulted in a dramatic elevation compared to all other groups (panel B).

### Effects on ET-1 and big ET-1

**Fig. 10:**
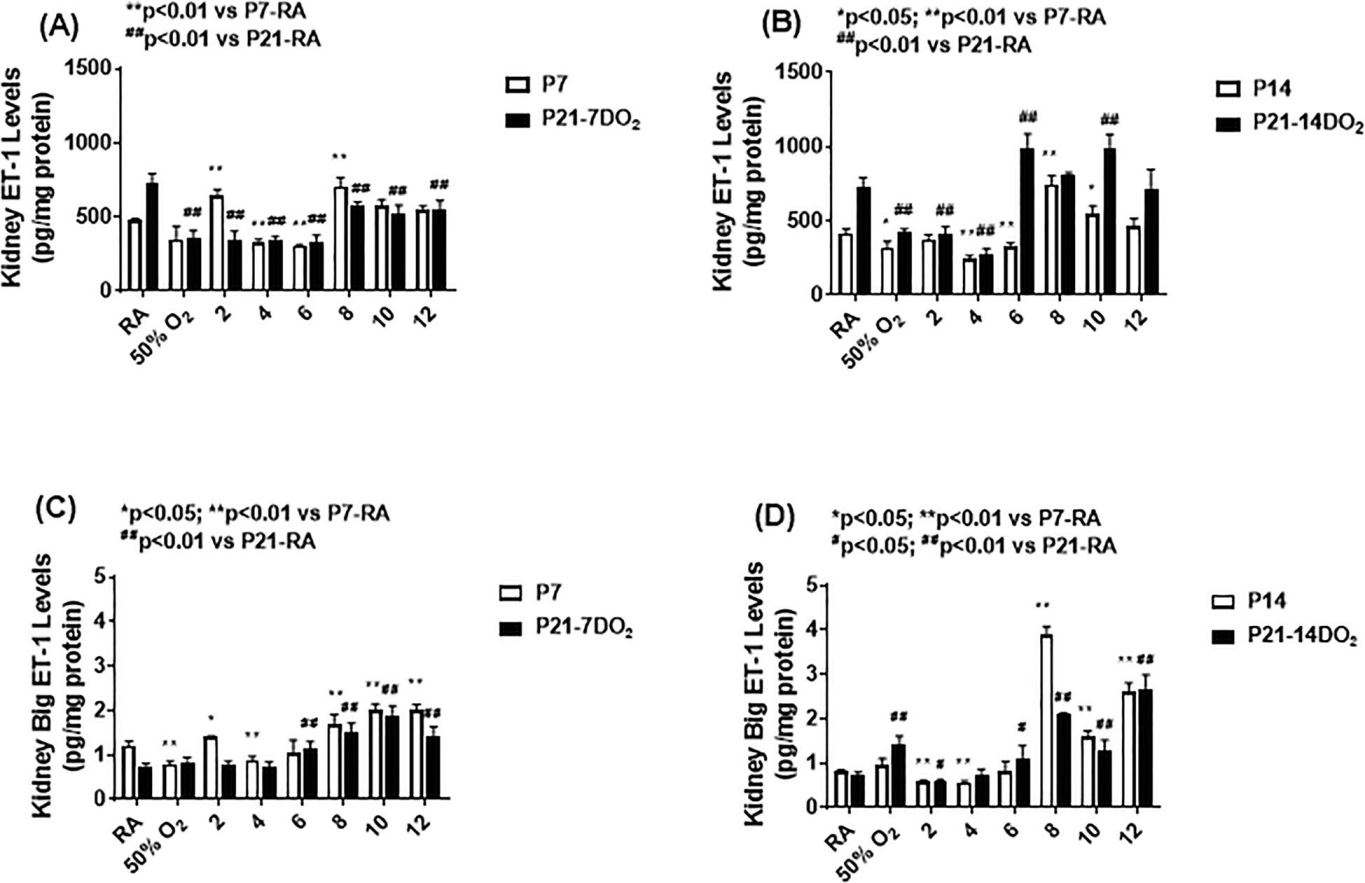
Effects of hyperoxia and incremental IH episodes on ET-1 (panels **A** and **B**) and big ET-1 (panels **C** and **D**) in the kidney homogenates from neonatal rats at P7 or P14 (open bar) and P21 (solid bar). Panels **A** and **C** are groups exposed from P0-P7 and P0-P7 with recovery/reoxygenation for 14 days from P7-P21. Panels **B** and **D** are groups exposed from P0-P14 and P0-P14 with recovery/reoxygenation for 7 days from P14-P21. Groups are as described in Fig. 1. Data are presented as mean ± SEM (*n* = 6 samples/groups)

**Fig. 11:**
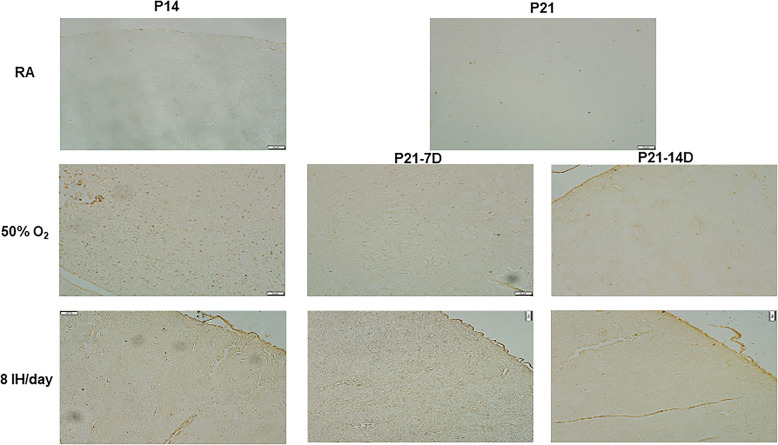
Representative immunohistochemistry staining of ET-1 in the kidneys in response to hyperoxia and IH episodes (8 episodes/day) at P14 and P21. P21–7D groups were exposed to hyperoxia or neonatal IH from P0-P7, then placed in RA from P7-P21. P21–14D groups were exposed to hyperoxia or neonatal IH from P0-P14, then placed in RA from P14-P21. Images are 20X magnification (scale bar is 50 μm)

**Fig. 12:**
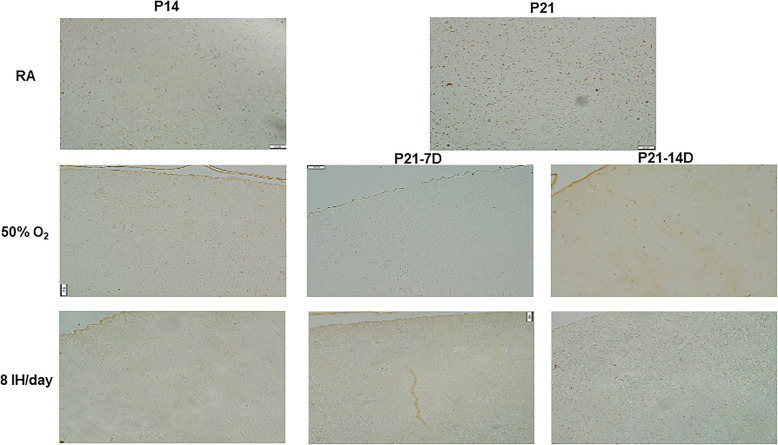
Representative immunohistochemistry staining of ET-2 in the kidneys in response to hyperoxia and IH episodes (8 episodes/day) at P14 and P21. P21–7D groups were exposed to hyperoxia or neonatal IH from P0-P7, then placed in RA from P7-P21. P21–14D groups were exposed to hyperoxia or neonatal IH from P0-P14, then placed in RA from P14-P21. Images are 20X magnification (scale bar is 50 μm)

Figure 10 shows the effects of hyperoxia and neonatal IH on ET-1 (panels A and B), and big ET-1 (panels C and D) levels in the kidney homogenates. Exposure for 7 days showed a decline in ET-1 with hyperoxia and 2–6 neonatal IH episodes/day. An opposite increase was seen with 8–12 IH episodes/day. The same effects persisted during the recovery/reoxygenation period (panel A). Exposure for 14 days resulted in the same response pattern with reductions from 0 (hyperoxia) to 6 IH episodes/day (except 2/day) and elevations with 8 and 10 IH episodes/day. The same effects persisted during the recovery/reoxygenation period (panel B). The effects of hyperoxia or neonatal IH on big ET-1 mirrored those on ET-1. Exposure for 7 days showed reductions with hyperoxia and 4 IH episodes/day, but increases with 2, 8, 10, and 12 episodes/day. During recovery/reoxygenation, elevations were noted with 6–12 episodes/day (panel C). Exposure for 14 days showed reductions with 2 and 4 IH episodes/day, and elevations with 8–12 IH episodes/day, peaking with 8 episodes/day. During recovery/reoxygenation elevations were noted with hyperoxia and 6–12 IH episodes/day, while reductions occurred with 2/day (panel D). Figure 11 shows the immunoreactivity of ET-1 in the renal cortex. ET-1 was not appreciably expressed in the renal cortex at P14 or P21. However, exposure to hyperoxia and 8 IH episodes/day increased ET-1 expression with and without recovery in RA. Table 1 shows the highest intensity of ET-1 immunoreactivity occurred with all hyperoxia and neonatal IH groups regardless of recovery/reoxygenation. Figure 12 shows that ET-2 immunoreactivity was highly expressed at P21 in the RA samples. Exposure to hyperoxia for 14 days decreased ET-2 compared to RA. Recovery from hyperoxia for 14 days further reduced ET-2, but recovery from hyperoxia for 7 days increased it. Table 1 shows that ET-2 was increased during and post hyperoxia and neonatal IH, except in the group recovering from 8 IH episodes/day for 14 days.

### Effects of ET receptors

**Fig. 13:**
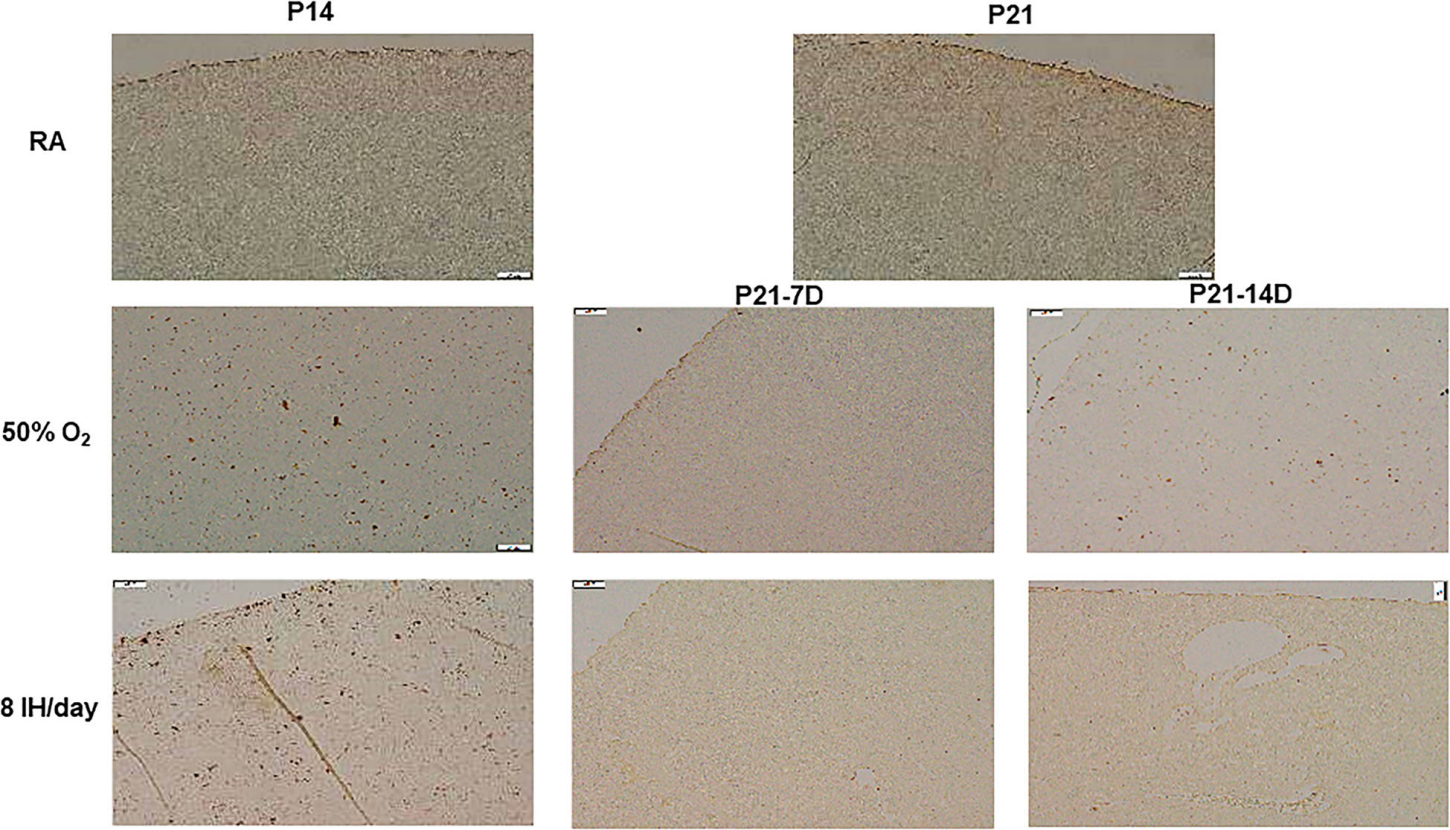
Representative immunohistochemistry staining of ET_A_R in the kidneys in response to hyperoxia and IH episodes (8 episodes/day) at P14 and P21. P21–7D groups were exposed to hyperoxia or neonatal IH from P0-P7, then placed in RA from P7-P21. P21–14D groups were exposed to hyperoxia or neonatal IH from P0-P14, then placed in RA from P14-P21. Images are 20X magnification (scale bar is 50 μm)

**Fig. 14:**
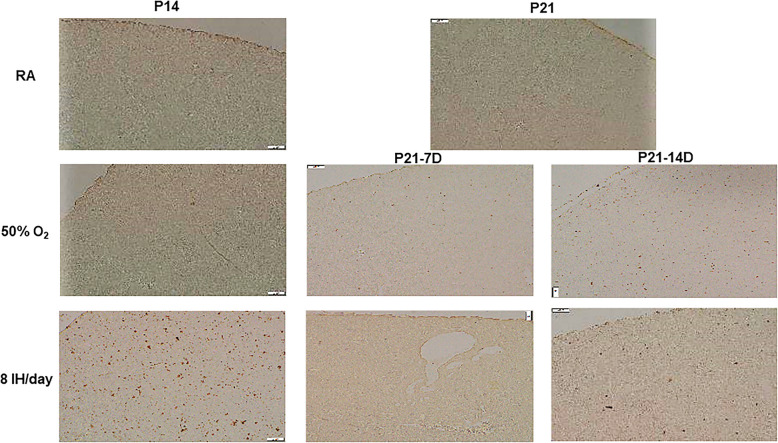
Representative immunohistochemistry staining of ET_B_R in the kidneys in response to hyperoxia and IH episodes (8 episodes/day) at P14 and P21. P21–7D groups were exposed to hyperoxia or neonatal IH from P0-P7, then placed in RA from P7-P21. P21–14D groups were exposed to hyperoxia or neonatal IH from P0-P14, then placed in RA from P14-P21. Images are 20X magnification (scale bar is 50 μm)

Figures 13 and 14 demonstrates the immunoreactivity of ET_A_R and ET_B_R, respectively in the renal cortex. ET_A_R was robustly elevated in the cortex at P14 and P21. Hyperoxia increased ET_A_R with modest reductions during the recovery/reoxygenation periods. Similarly, exposure to 8 IH episodes/day caused a robust increase in ET_A_R expression, an effect that remained sustained during recovery/reoxygenation, but was lower that exposure for P14 (Fig. 13). Similar elevations in ET_B_R expression were seen with 8 IH episodes/day with and without recovery/reoxygenation, although the levels declined during recovery/reoxygenation compared to exposure for 14 days (Fig. 14). Table 1 shows that elevations in ET_A_R with 14 days of hyperoxia and neonatal IH (8 episodes/day). However, the levels declined during recovery from hyperoxia, but not during recovery from 7 days of neonatal IH. In contrast, ET_B_R expression declined with hyperoxia and subsequent recovery/reoxygenation compared to RA, and was elevated with 8 neonatal IH episodes/day. However, the levels declined during the recovery/reoxygenation period.

### Effect on HIF_1α_

**Fig. 15:**
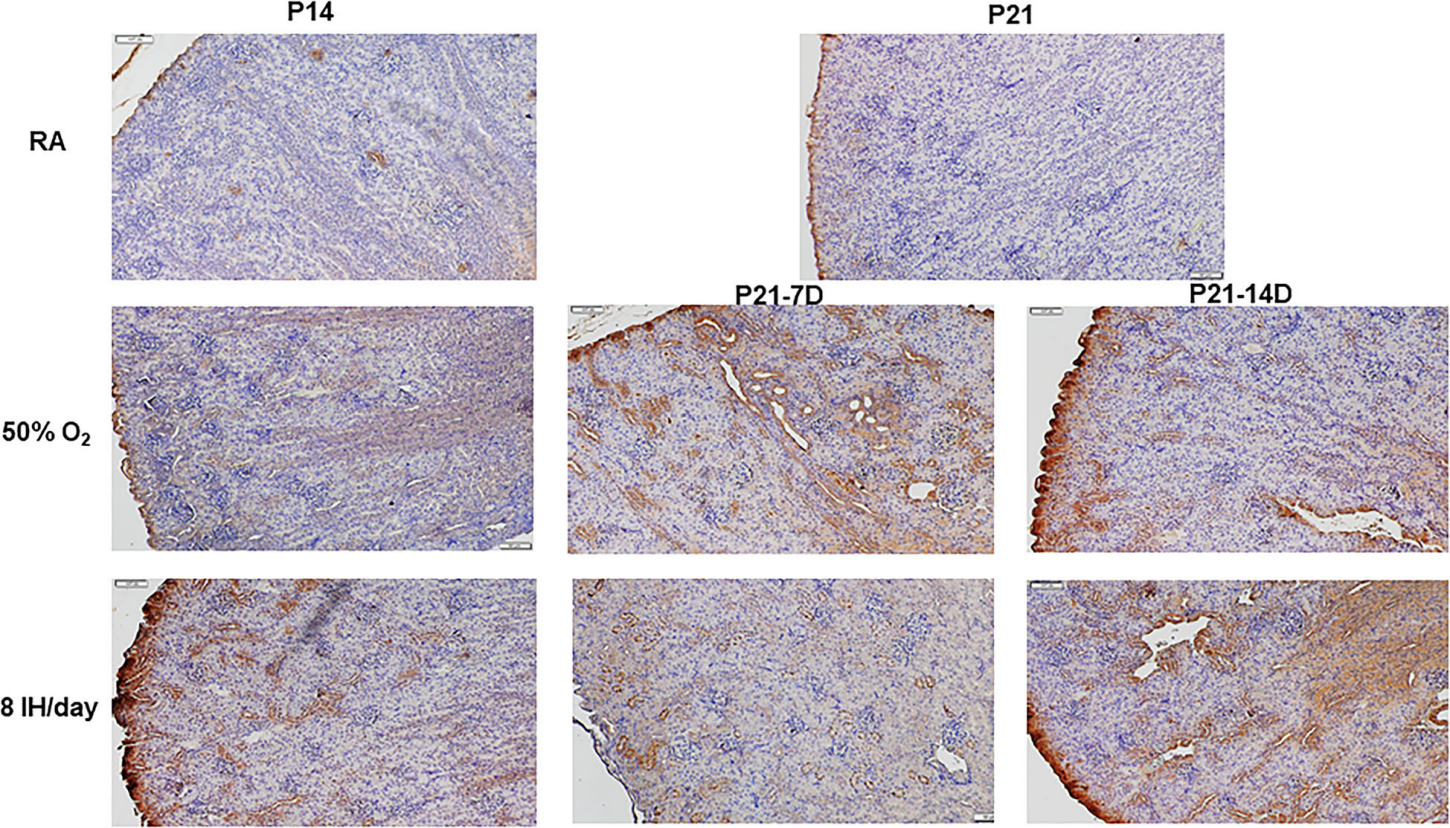
Representative immunohistochemistry staining of HIF_1α_ in the kidneys in response to hyperoxia and IH episodes (8 episodes/day) at P14 and P21. P21–7D groups were exposed to hyperoxia or neonatal IH from P0-P7, then placed in RA from P7-P21. P21–14D groups were exposed to hyperoxia or neonatal IH from P0-P14, then placed in RA from P14-P21. Images are 20X magnification (scale bar is 50 μm)

HIF_1α_ immunoreactivity (brown) in response to hyperoxia and neonatal IH in the kidneys is presented in Fig. 15. Sections were counterstained with hematoxylin (blue). There was minor staining in the P14 and P21 RA sections which predominated in the renal capsule. Compared to RA, no major differences were noted with exposure to 50% O_2_ for 14 days. In contrast, robust staining was noted in the renal capsule and cortex with exposure to 8 neonatal IH episodes/day for 14 days. Similar responses were noted in the groups recovering from 7 and 14 days of hyperoxia and neonatal IH. Table 1 shows the corresponding quantitative analysis of HIF_1α_ expression.

### Effect on apoptosis

**Fig. 16:**
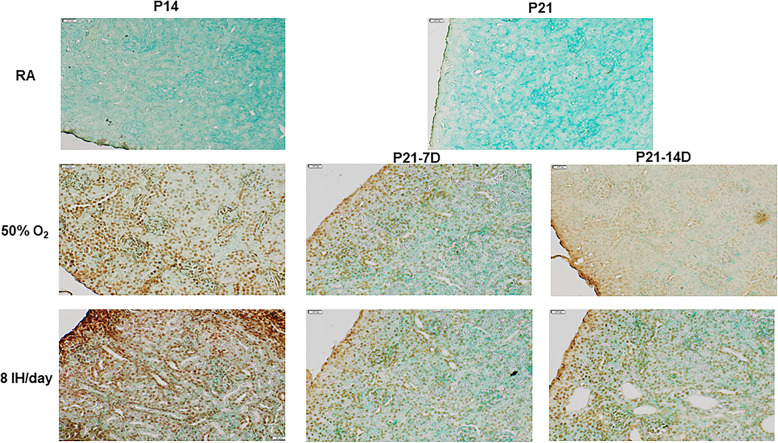
Representative images of TUNEL stained kidney sections counterstained with Methyl Green, in response to hyperoxia and IH episodes (8 episodes/day) at P14 and P21. P21–7D groups were exposed to hyperoxia or neonatal IH from P0-P7, then placed in RA from P7-P21. P21–14D groups were exposed to hyperoxia or neonatal IH from P0-P14, then placed in RA from P14-P21. Positive apoptosis is indicated by dark brown signal. Green indicates non-reactivity/negative. Images are 40X magnification (scale bar is 20 μm)

TUNEL stained kidney sections counterstained with Methyl Green are presented in Fig. 16. Positive apoptosis is indicated by a dark brown signal and green indicates non-reactive or negative reactivity. Groups exposed to RA were negative while groups exposed to 50% O2 and 8 IH episodes/day for 14 days showed strong reactivity in the renal capsule and cortex. Groups recovering from hyperoxia and neonatal IH showed less intense reactivity, but positive staining persisted throughout the renal cortex. Table 1 shows the corresponding quantitative analysis.

## Discussion

This study showed that hyperoxia as well as increasing episodes of neonatal IH can cause detrimental and irreversible damage to the developing kidney, evidenced by increasing damage severity, apoptosis, and necrosis as a function of neonatal IH episodes. The levels of vasoconstrictors Ang II, ACE, ACE-2, ET-1 increased even during the recovery/reoxygenation phases of the study, regardless of the length of exposure. This indicates that neonatal IH is indeed associated with biomarkers of hypertension and in part, may contribute to kidney damage, thus proving our hypothesis. ACE-2 levels involved in vasodilation also increased to counter the vasoconstrictive/damaging pathways, but to a lesser degree and with a limited ability to compensate, as the frequency of neonatal IH increased resulting in preponderance of the vasoconstrictive effects. In this model, we examined rat pups at days 7, 14, and 21 of life in order to further examine the effects of neonatal IH at various stages of renal development. At 0–14 days of life, rat pups correlate to the neonatal period, where they mainly consume their mothers’ milk [29]. However, from days 14–21, the rat pups gradually begin to consume solid food, go through the weaning process, and are fully weaned at P21. Consumption of solid food also aligns with salt intake. Nephrogenesis is completed on postnatal day 8 [30]. This transition may partly explain why the rat pups at P7 are more sensitive to the effects of neonatal IH resulting in higher ACE and lower ACE-2 levels compared to P14 and P21. To our knowledge, this is the first study to show low levels of renal ACE-2 in the suckling period. These developmentally low levels may account for an inability to mount a compensatory and protective ACE-2 response to counteract ACE levels.

The histopathology results demonstrated that both chronic hyperoxia and neonatal IH are detrimental to the developing kidney, but neonatal IH produced the worst damage. The morphometric analysis supported this finding and a notable increase in kidney size was found in the hyperoxia samples as compared to the controls, possibly due to increased hemorrhage and possible hypertrophy [31]. Animal models have demonstrated enlarged renal corpuscles and subsequent decreased nephron number in kidneys during periods of hyperoxia [32, 33]. Previous studies have also shown that neonatal hyperoxia exposure results in impaired nephrogenesis, affecting the nephrogenic zone width and glomerular diameter as well as increased apoptotic cell count [34]. There are sparse studies on the impact of neonatal IH on kidney damage and biomarkers of hypertension. A study involving 8-week old mice with obstructive sleep apnea found that IH caused kidney injury accompanied by glomerular hypertrophy, mesangial matrix expansion, increased expression of glomerular growth factors and an increased cellular apoptosis [35]. In our study, shorter neonatal IH exposure time with a longer recovery/re-oxygenation time resulted in more renal damage than longer exposures with shorter recovery. This may be due to upregulation of mechanisms involved in reperfusion injury. The shorter recovery/reoxygenation time may not be sufficient to initiate those responses. It seems reasonable that a longer IH exposure would require comparatively longer recovery/reoxygenation. The severity of kidney damage was directly correlated with neonatal exposure time with the worst damage seen in the 12 episodes/day group, where severe hemorrhage and necrosis are demonstrated. The findings in this study prove that hyperoxia and more importantly, neonatal IH can both cause irreversible damage to the kidney structure regardless of recovery/re-oxygenation [31, 36]. These are findings are consistent with necrosis that can later lead to the development of chronic kidney disease, supporting our hypothesis.

Malondialdehyde (MDA) is produced by oxidation of polyunsaturated fatty acids [37], and is a major determinant of lipid peroxidation [38]. Our study showed that MDA was significantly higher in the groups recovering from all IH episodes, particularly 8–12 episodes. This finding provides evidence for reperfusion injury and suggests that the immature kidney is highly susceptible to IH-induced lipid peroxidation and cannot sustain more than 6 episodes per day. Given the numerous IH episodes reported to occur in preterm infants during the first few weeks of life [4], this may explain, in part, the higher risk for developing AKI [39]. Monitoring MDA levels may be a useful biomarker to identify infants at risk.

Neonatal IH also caused high levels of Ang II and ACE, with a plateau seen at 8 episodes per day followed by a decline at 10–12 episodes. This may indicate that 8 IH episodes/day is the maximum number that the developing kidney can sustain. The decline with 8–12 episodes suggest either that the tissue is deteriorating and unable to mount a response, or that there is a bi-phasic response with the shift from predominant hyperoxia (less IH episodes) to predominant hypoxia (more IH episodes). The immunoreactivity results of ACE analysis support these findings. Ang II, ACE and ACE-2 are all part of the RAAS system, and serve important functions for the preservation of the kidney. While Ang II and ACE are involved in vasoconstriction and apoptosis, ACE-2 works to counter this effect and balance the system with vasodilation [40–46]. Ang II is strongly associated with re-perfusion injury and AKI, via reactive oxygen species (ROS) production. Our data demonstrated a strong correlation with increased IH episodes and renal Ang II and ACE production, suggesting renal vasoconstriction and oxidative damage as seen in AKI [42, 43, 46].

Another important finding was that the ACE response was more robust in the P7 and P14 animals, while the P21 animals demonstrated a stronger ACE-2 response, suggesting that ACE-2 is activated with advancing renal development and may be protective, and take precedence over ACE. ACE-2 has the primary function of degrading Ang II and opposes the effect of ACE [37–39]. However, this effect may be overridden in neonatal IH as shown in the more mature P14 and P21 rats exposed to 8 IH episodes/day. A study involving ACE knockout mice, found that despite unopposed ACE and Ang II activity, kidney function and renal development was normal [47]. However, in periods of RAAS activation and kidney stress ACE-2 becomes a more vital for preventing kidney damage [45, 46]. Similar to ACE, the efficacy with which the kidney can incite the release of ACE-2 during stress reaches a threshold after which it wanes. Multiple studies have supported this finding in patients with CKD undergoing dialysis having lower levels of ACE-2 compared to pre-dialysis patients [47, 48]. The ACE-2 pathway may be more effective as reno-protective in AKI and early CKD [48–50].

The ACE/ACE-2 ratios were higher with shorter exposure time, peaking with 6 IH episodes/day. Longer exposure time resulted in ACE-2 overriding ACE and resulting in balanced levels, except in the group exposed to 8 IH episodes. Studies found that ACE/ACE-2 ratios were significantly higher in patients with hypertension than in those without [51]. Other studies in rats showed higher ACE mRNA compared to ACE-2 mRNA leading to elevated ACE/ACE-2. Those studies confirm that the ACE-Ang II axis was dominant in severe kidney injury [52]. This balance of ACE and ACE-2 is important in maintaining the equilibrium in the kidney, and using these pathways the neonatal kidney appears able to compensate for stress up to a point. However, when threshold is reached, despite subsequent recovery, the damage is irreversible and these pathways are no longer able to function properly, thus leading to CKD.

The effects of increasing IH episodes on ET-1 were interesting. Short term exposure resulted in elevated ET-I levels in all groups exposed to 8–12 IH episodes, while long-term exposure resulted in an earlier rise with 6–12 IH episodes. Given that ET-1 is a potent vasoconstrictor, these findings provide clear support that neonatal IH produces hypertension, with no resolution during the recovery/reoxygenation phase, suggesting a permanent effect. Our findings confirm those of others which showed that ET-1 is activated during periods of stress [29, 53–56] such as with hyperoxia and intermittent hypoxia. ET-1 acting via the ET receptors is rapidly up-regulated in the kidney by ischemia and has been implicated in renal inflammation and hypertension [57–60]. The endothelin system has also been largely implicated as being involved in CKD [55, 56]. Big ET-1 is the precursor to ET-1 via proteolytic action, and has minimal biological function, but is able to bind to endothelin receptors with lower affinity [56–58]. The concurrent elevation in big ET-1 provides further evidence of a vasoconstrictor response, as this precursor is cleaved to form ET-1. This finding suggests that neonatal IH induces or promotes big ET-1 cleavage.

Compared to RA and hyperoxia, 8 neonatal IH episodes/day increased ET_A_R and ET_B_R immunoreactivity. ET_A_R and ET_B_R both serve as receptors for ET-1 but have opposing effects depending on the cell type, tissue type, or physiological situation [58–60]. ET_A_R has been implicated in vasoconstriction and may play a more active role in causing damage to the kidney when the organ is placed under stress [58, 59, 61–65]. ET_A_R cause an agonist effect on ET-1, and may prevent its degradation [58, 64, 65]. Conversely ET_B_R may promote ET-1 clearance and may be more involved in vascular dilatation [11, 58, 64–66]. Our findings demonstrated that ET_A_R increased with damage caused from hyperoxia and neonatal IH, while ET_B_R declined. Higher ET-1 and ET_A_R with 8–12 neonatal IH episodes/day were consistent with Ang II trends. The increasing severity of damage provide strong evidence that neonatal IH is involved in the pathogenesis of AKI in neonates. Medication which targets these pathways to promote vasodilation and inhibit vasoconstriction could help prevent AKI and subsequent CKD.

Hypoxia-inducible factor (HIF)_1α_ is a transcription factor that regulates the expression of numerous genes and activates various downstream signaling pathways, including erythropoietin production, angiogenesis, energy metabolism, and other related pathways, to facilitate cell adaptation to the anoxic environment [67]. Studies show that HIF_1α_ plays exerts a protective role in acute kidney injury [68]. In our study HIF_1α_ was higher during recovery/reoxygenation from hyperoxia, but also during and post neonatal IH, further indicating its renoprotective role. However, studies show that induction of HIF_1α_ was associated with reduced apoptosis in rat proximal tubular cells subjected to hypoxia and kidney tissues of mice after renal ischemia-reperfusion injury [69]. In our study, apoptosis was indeed higher in the hyperoxia group with lower HIF_1α_. However, this was not the case with exposure to 8 IH episodes, suggesting that frequent IH episodes may override the protective effect.

### Clinical implications

The neonatal IH model used in these experiments is clinically relevant. We previously demonstrated that the brief clustered IH pattern is more damaging than dispersed IH episodes [26]. Using complex mathematical modeling, DiFiore M, et al. [27] later confirmed our findings in preterm infants. Premature neonates, particularly soon after birth, are more vulnerable to the development of AKI and CKD [70]. Stress resulting from hyperoxia and neonatal IH may cause structural damage as well changes in the physiological pathways involved in the function of the kidney. The kidneys appear able to recover shorter numbers of IH episodes/per day due to the longer recovery time between episodes. However, when a threshold is reached due to more frequent IH episodes, resulting in decreased recovery time between episodes, the neonatal kidneys may no longer be able to a mount a compensatory response. This study provided a novel model of assessing the damage caused to the kidney by neonatal IH, which had not been previously assessed in a similar context.

## Conclusions

While the study has important clinical implications, there are limitations. For example, the rat model may not accurately reflect apnea experienced by ELGANs and periviable neonates who may be the most susceptible to the kidney damage [66, 71]. These neonates may possibly have different thresholds for compensatory responses. Additionally, we did not examine renal histopathology at P7. Nevertheless, the most damaging effects were noted in the groups exposed to 8 IH episodes/day, confirming that a critical number of daily IH episodes that may result in irreparable renal damage. Long term outcomes such as urinary biomarkers of kidney function and changes in blood pressure was not determined in adult rats, but could be the focus of future studies. Our study suggests that the critical number of IH episodes that the immature kidneys can sustain is 6, beyond which irreparable damage may occur. The Ang II and ET-1 pathways appear to be highly involved in IH-induced renal damage. Therapeutic targeting of these pathways may help decrease the risk for kidney injury in the developing neonate to prevent and/or treat neonatal AKI and CKD.

## Supplementary Information

**Additional file 1: Figure S1.** Experimental Design.

**Additional file 2: Figure S2.** Intermittent Hypoxia Paradigm.

## Data Availability

The datasets used and/or analyzed during the current study are available from the corresponding author on reasonable request.

## Abbreviations

ACE: Angiotensin converting enzyme
AKI: Acute kidney injury
Ang: Angiotensin
ANOVA: Analysis of variance
ARRIVE: Animal Research: Reporting of In Vivo Experiments
AVMA: American Veterinary Medical Association
CKD: Chronic kidney disease
ELGANs: Extremely low gestational age neonates
ELISA: Enzyme-linked immunosorbent assay
ET: Endothelin
ET_A_R: Endothelin A receptor
ET_B_R: Endothelin B receptor
FiO_2_: Fraction of inspired oxygen
H&E: Hematoxylin and eosin
HIF_1α_: Hypoxia-inducible factor-_1α_
ICU: Intensive care unit
IH: Intermittent hypoxia
IHC: Immunohistochemistry
MDA: Malondialdehyde
NBF: Neutral buffered saline
NO: Nitric oxide
P0: Postnatal day 0
P14: Postnatal day 14
P21: Postnatal day 21
P21–14DO_2_: Neonatal IH for 14 days and room air for 7 days
P21–7DO_2_: Neonatal IH for 7 days and room air for 14 days
P7: Postnatal day 7
PBS: Phosphate buffered saline
RA: Room air
RAS: Renin-angiotensin system
SUNY: State University of New York
